# Supplementary material for: Spatial genetic structure of 2009 H1N1 pandemic influenza established as a result of interaction with human populations in mainland China
Source: PLoS One. 2023 May 17;18(5):e0284716. doi: 10.1371/journal.pone.0284716 (PMC10191359; doi:10.1371/journal.pone.0284716)

Temporal phylogeny

HA (n = 413)

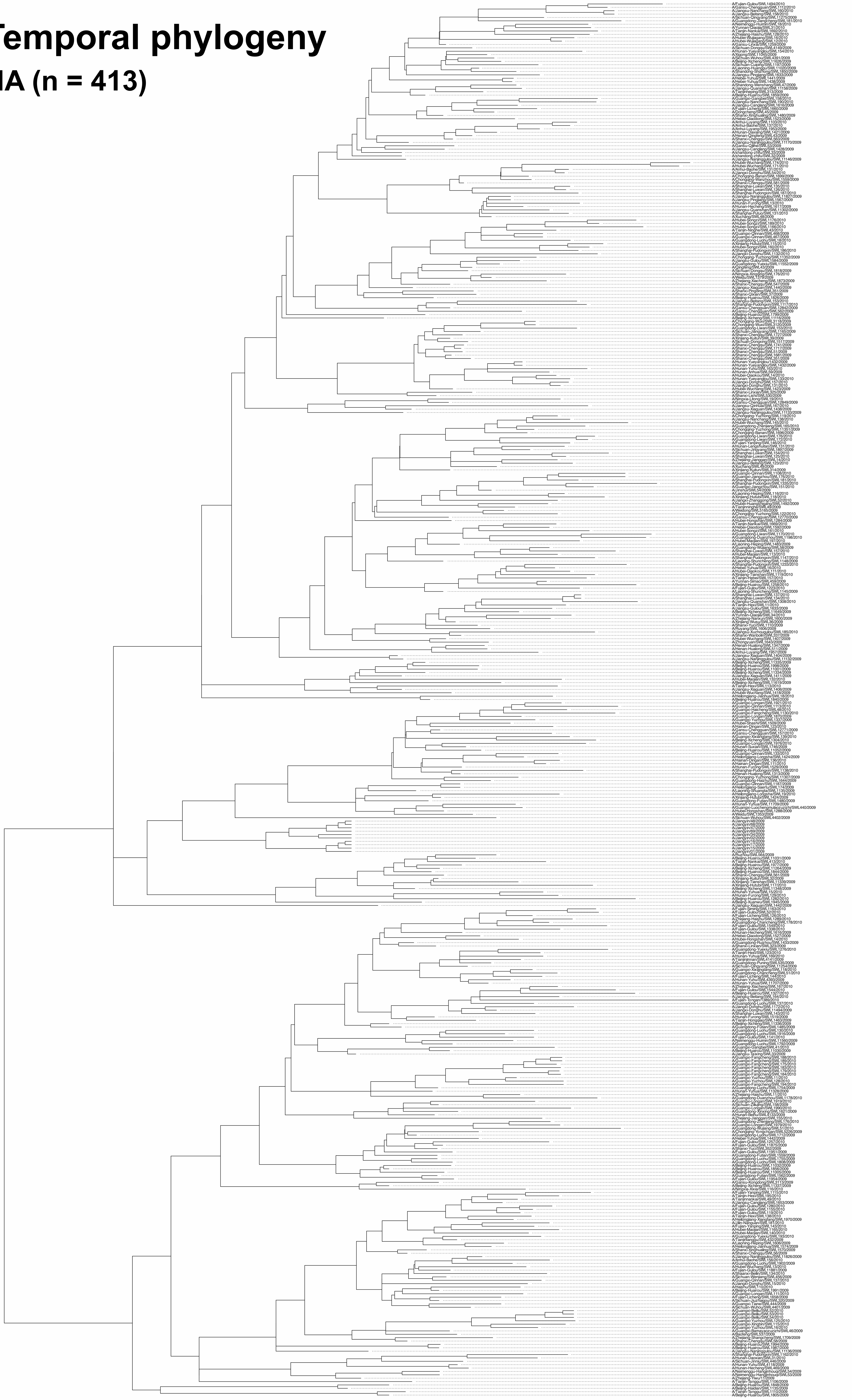

Supplement: S1 Fig — (PDF) [file pone.0284716.s001.pdf]
