# Supplementary figures and images for: Spatial genetic structure of 2009 H1N1 pandemic influenza established as a result of interaction with human populations in mainland China
Source: PLoS One. 2023 May 17;18(5):e0284716. doi: 10.1371/journal.pone.0284716 (PMC10191359; doi:10.1371/journal.pone.0284716)

## NA (n = 406)

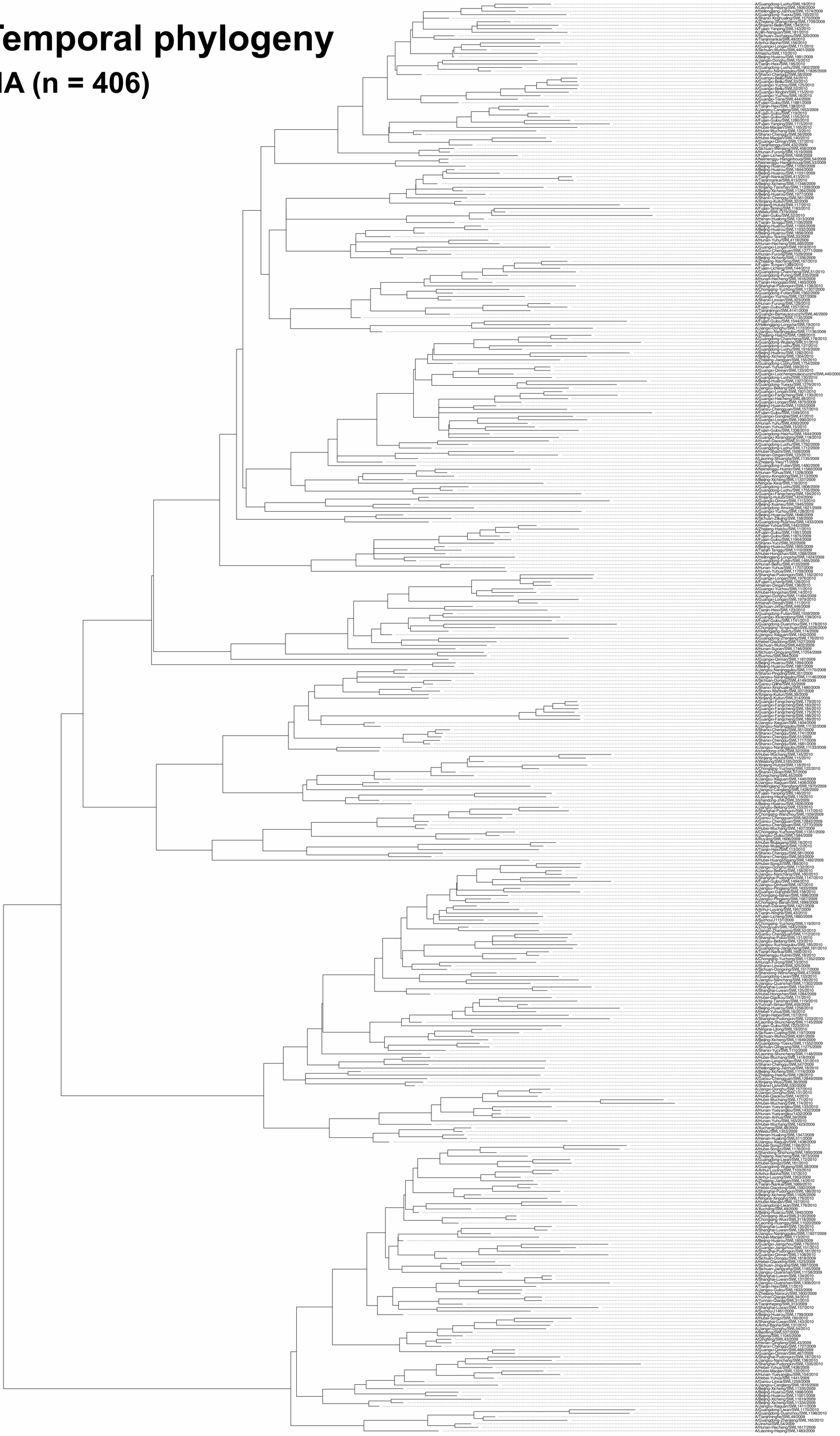

Supplement: S2 Fig — (PDF) [file pone.0284716.s002.pdf]

## HA (n = 413)

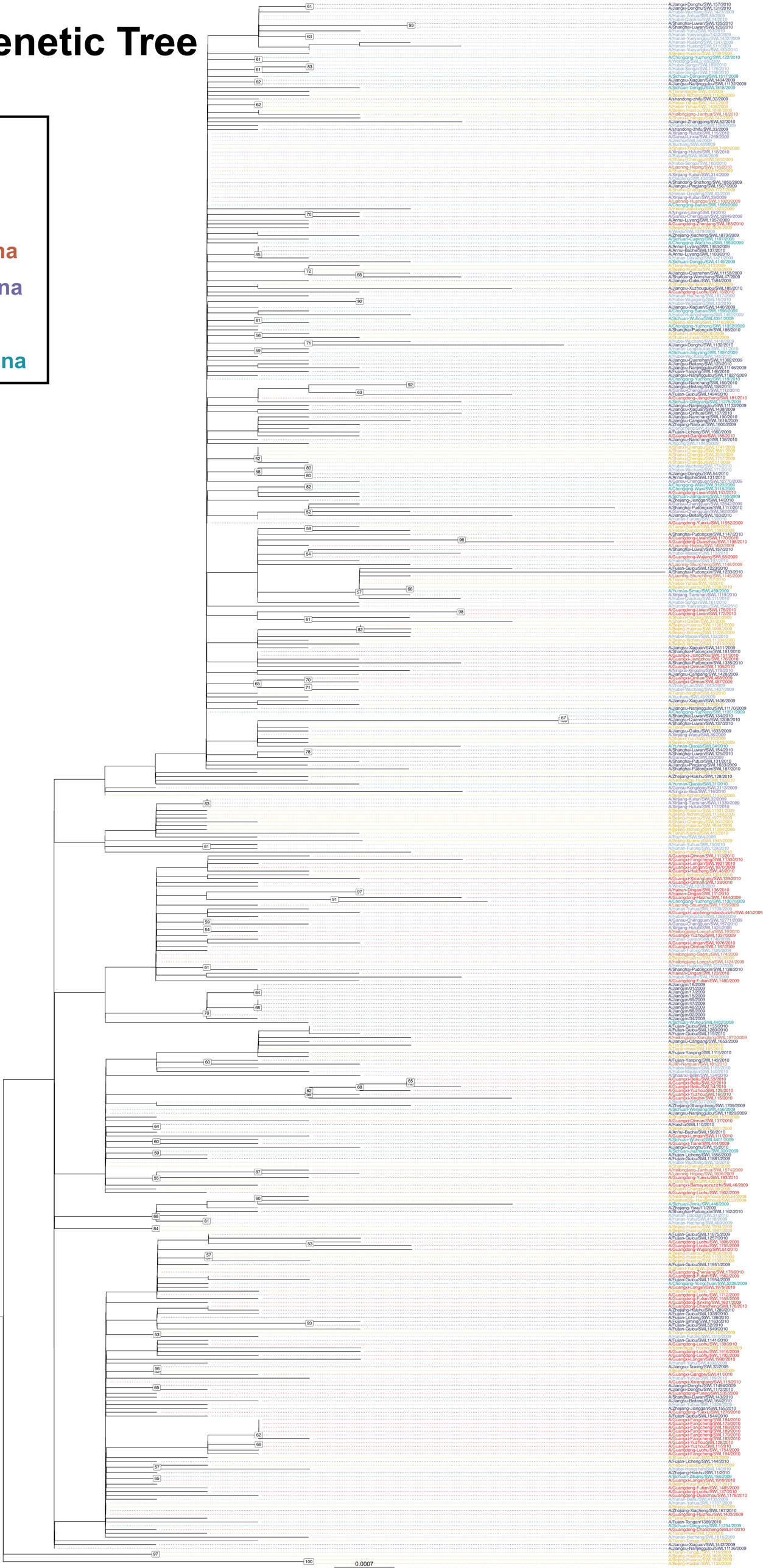

Supplement: S3 Fig — Internal nodes with a bootstrap support of 50% or greater (1,000 replications) are indicated. (PDF) [file pone.0284716.s003.pdf]

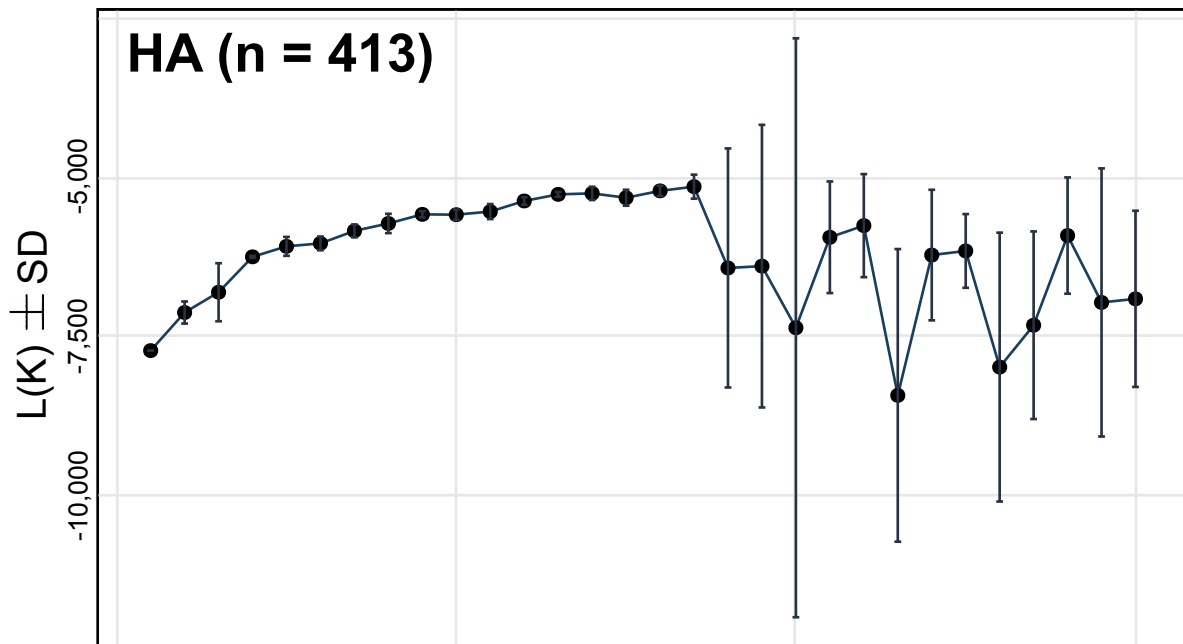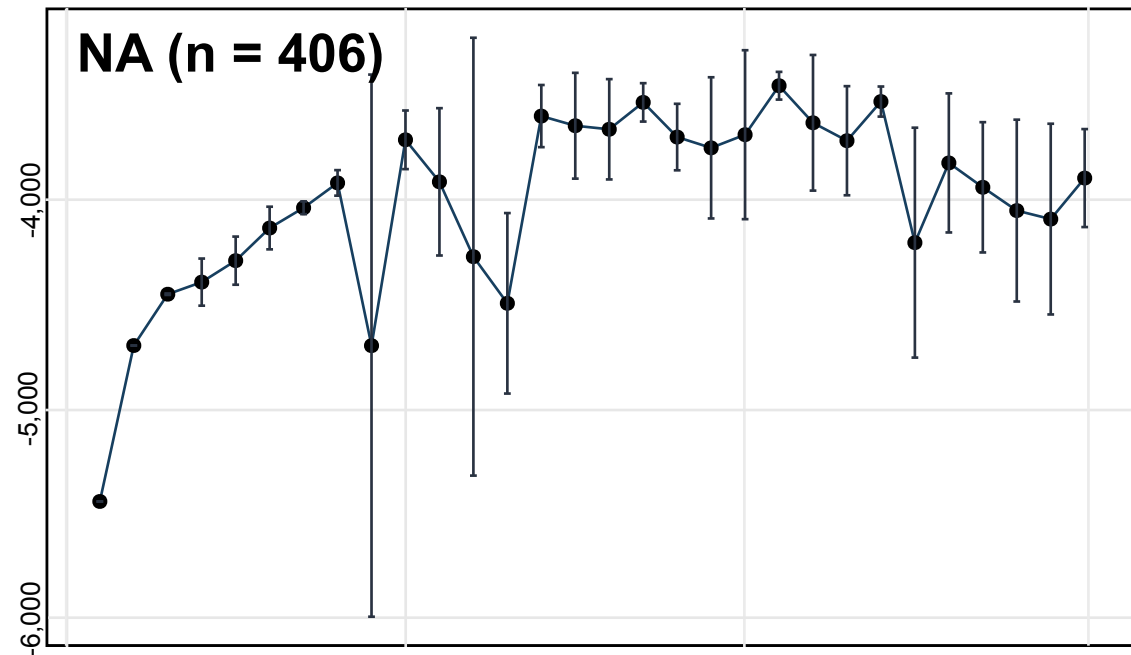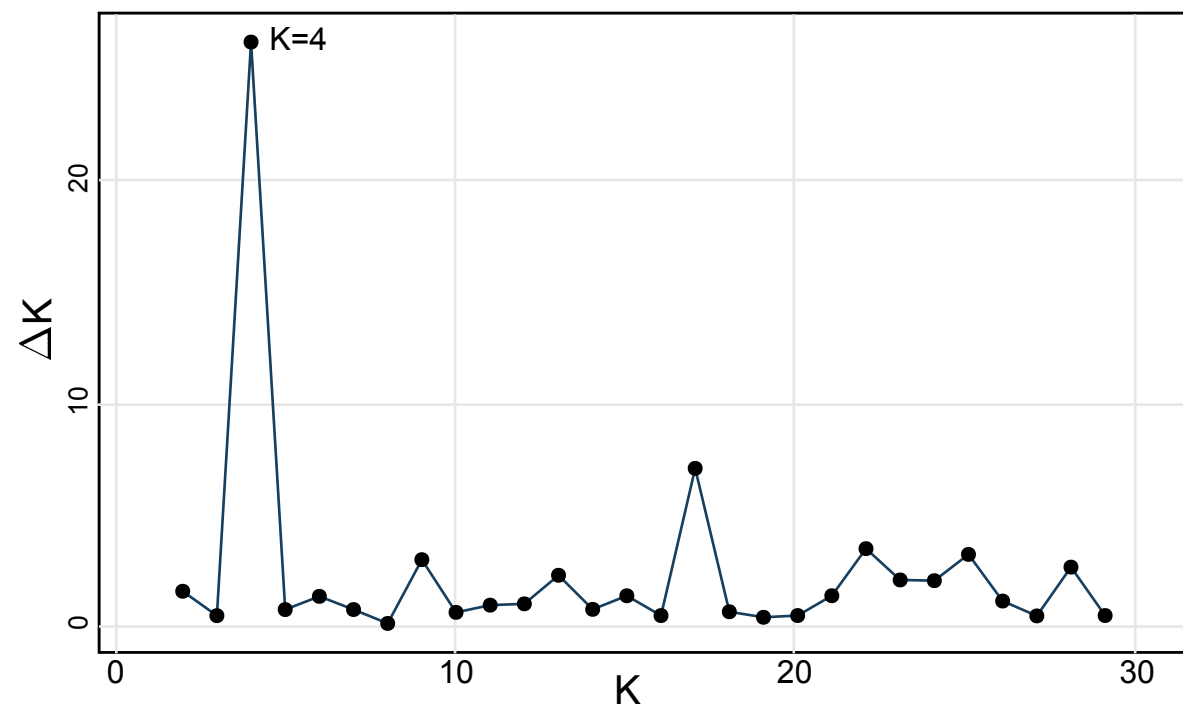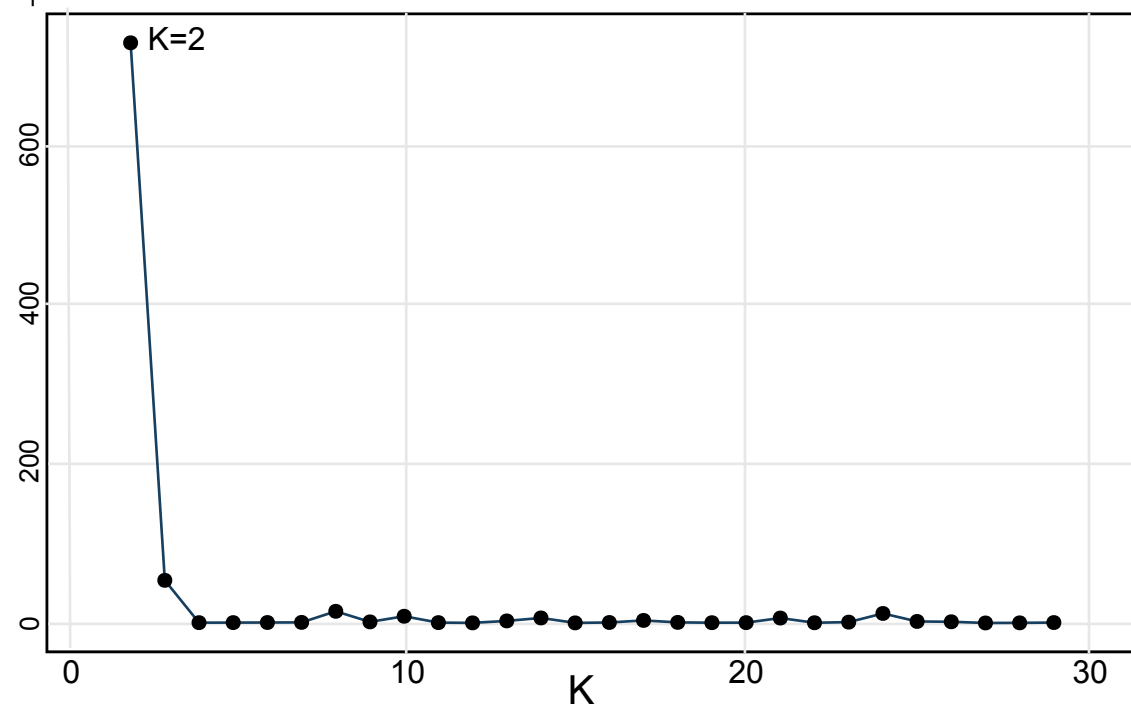

Supplement: S5 Fig — (PDF) [file pone.0284716.s005.pdf]

HA (n = 413)

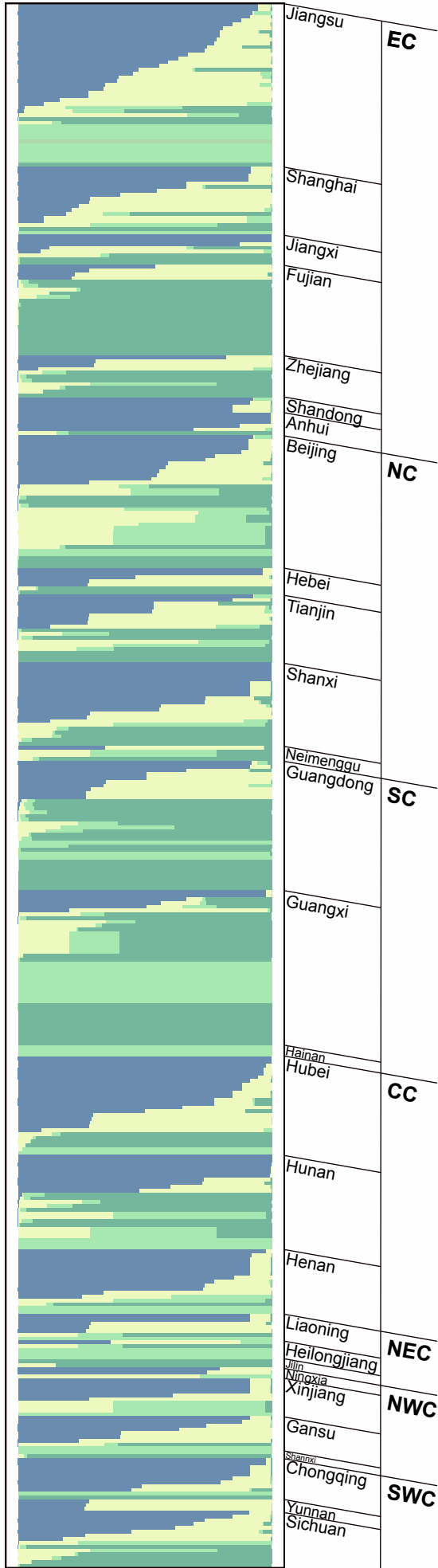

NA (n = 406)

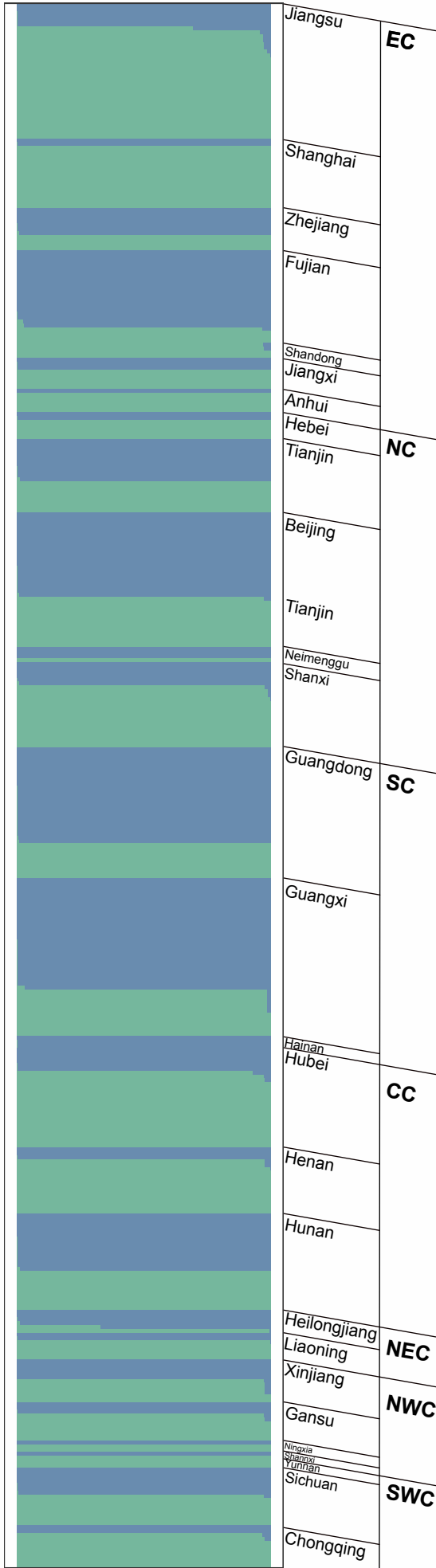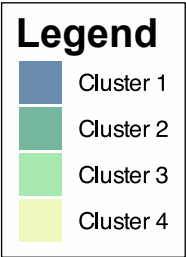

Supplement: S6 Fig — (PDF) [file pone.0284716.s006.pdf]

A. Shandong

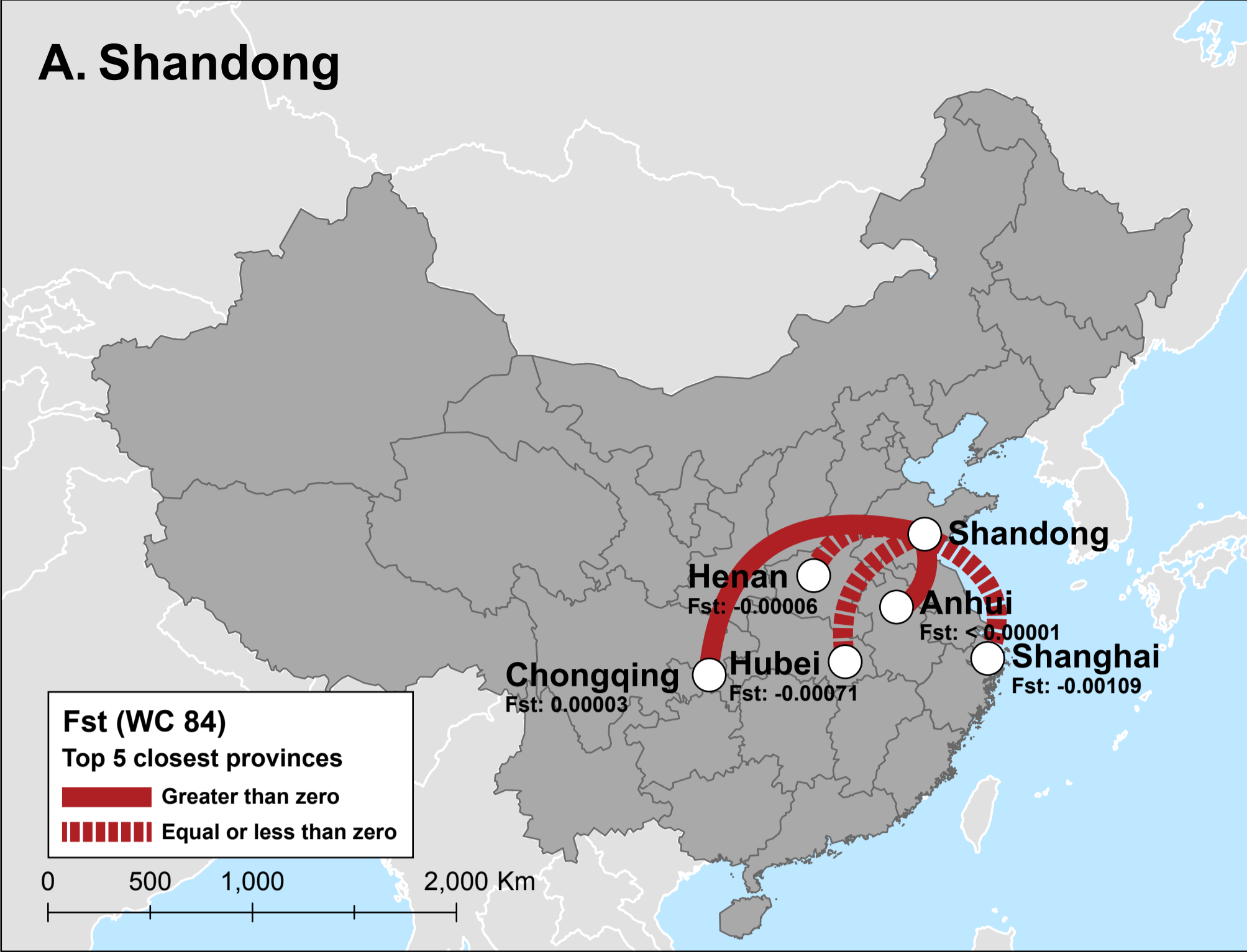

B. Guangdong

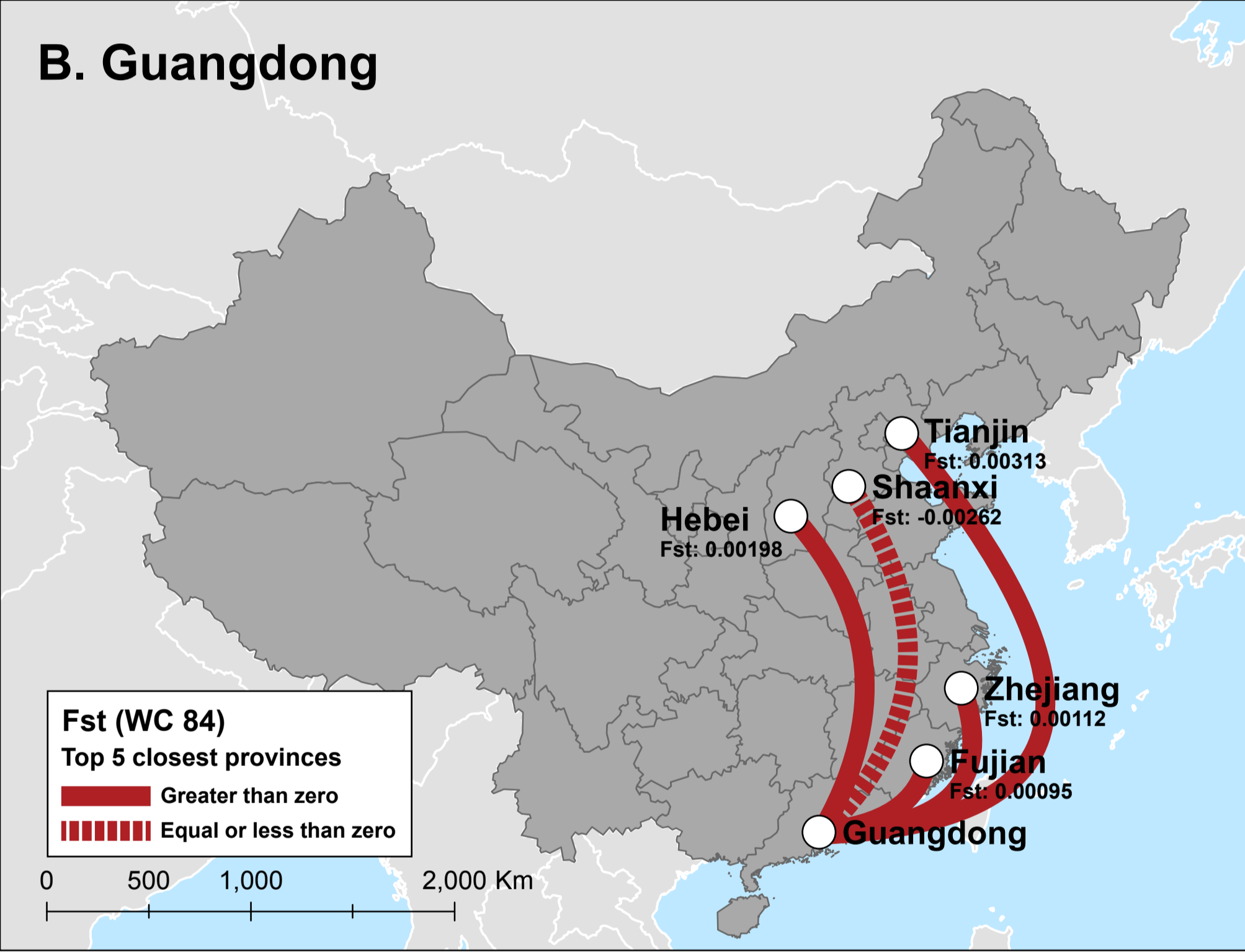

C. Hainan

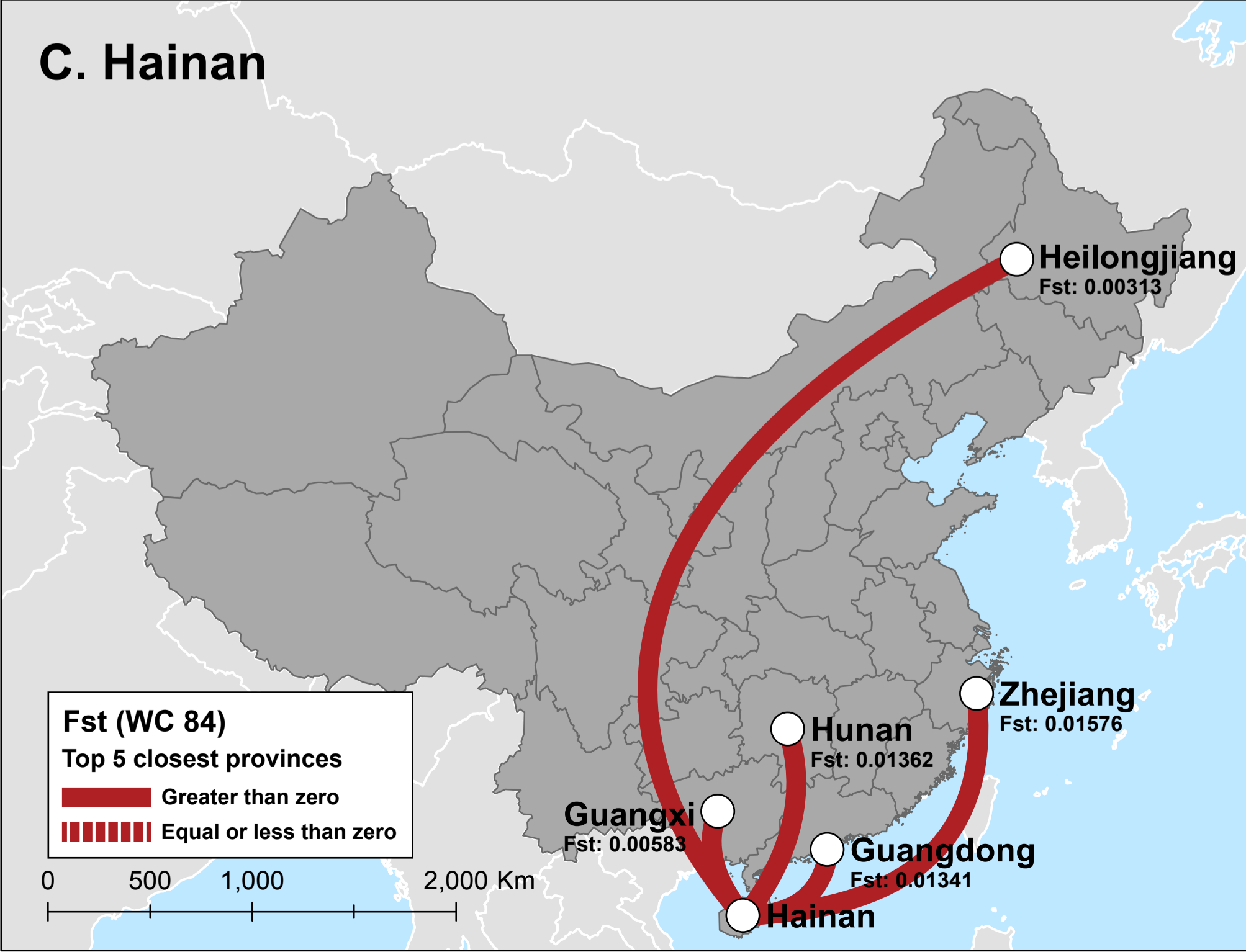

Supplement: S7 Fig — (PDF) [file pone.0284716.s007.pdf]
