# Supplementary material for: Spatial genetic structure of 2009 H1N1 pandemic influenza established as a result of interaction with human populations in mainland China
Source: PLoS One. 2023 May 17;18(5):e0284716. doi: 10.1371/journal.pone.0284716 (PMC10191359; doi:10.1371/journal.pone.0284716)

ML Phylogenetic Tree

NA (n = 406)

- Central China
- East China
- North China
- Northeast China
- Northwest China
- South China
- Southwest China

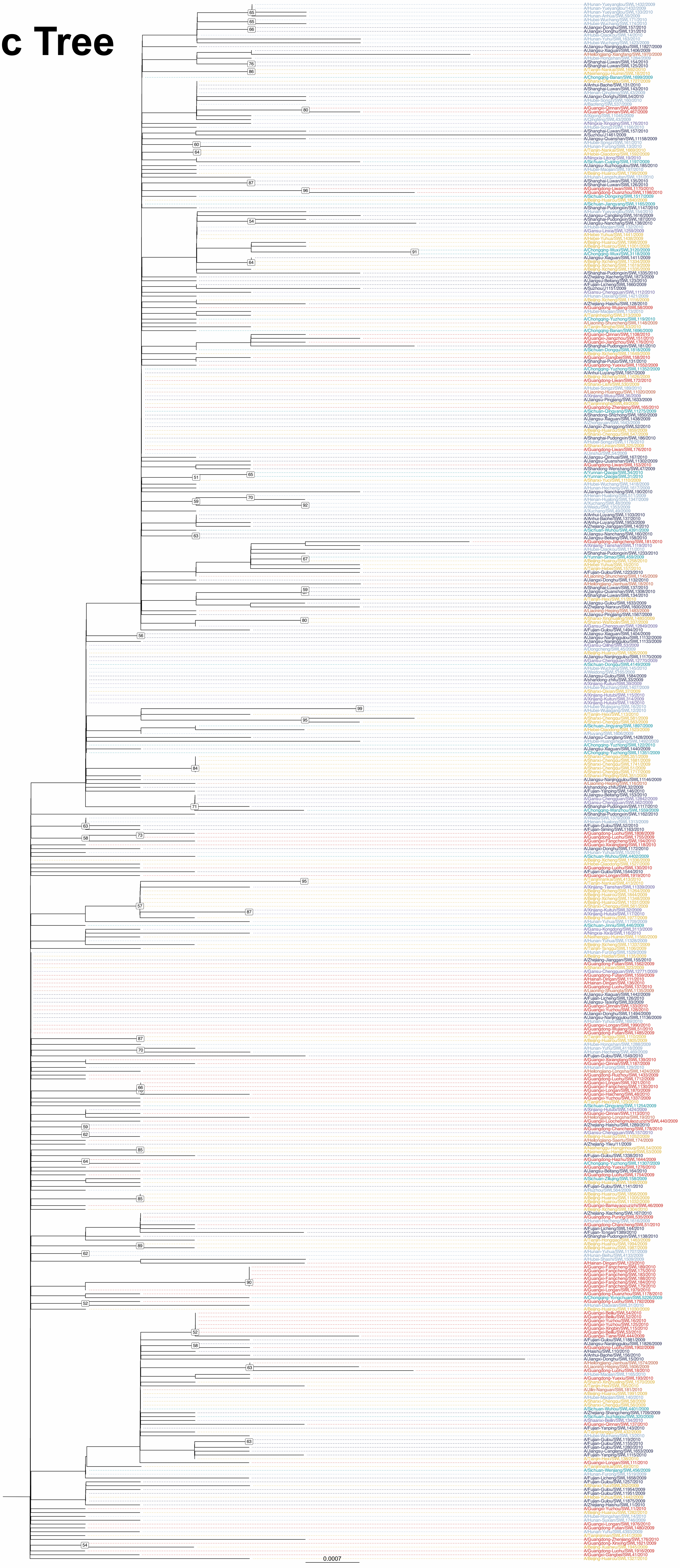

Supplement: S4 Fig — Internal nodes with a bootstrap support of 50% or greater (1,000 replications) are indicated. (PDF) [file pone.0284716.s004.pdf]
