# Supplementary material for: Spatial genetic structure of 2009 H1N1 pandemic influenza established as a result of interaction with human populations in mainland China
Source: PLoS One. 2023 May 17;18(5):e0284716. doi: 10.1371/journal.pone.0284716 (PMC10191359; doi:10.1371/journal.pone.0284716)
Supplement: Attachment — Submitted filename: Response to Reviewers.docx [file pone.0284716.s013.docx]

**Dear Dr. Arenas and Editors,**

Thank you for providing us the opportunity to revise the manuscript. We appreciate the time and effort that Dr. Arenas and editors have dedicated to providing valuable feedback. Please find the revised manuscript and our answers based on the reviewer’s comments below.

**Editor: Copyright of Figure 1, 5 and 6**

We generated our figures using the world administrative boundaries shapefile (vector format for spatial data) obtained from the World Bank. The source of the shapefile is noted in the revised manuscript from line 246 to 247, and their license information and policy can be found in the link below:

<https://datacatalog.worldbank.org/public-licenses?fragment=cc>

**Main comment 1**

**Comment:** I missed an analysis of genetic recombination in these data. Recombination can affect genetic diversity, genetic structure and also the phylogenetic tree reconstructions and inferences derived.

**Answer:** Recombination is one of the main drivers of the evolution of influenza A viruses, particularly with regard to the process of reassortment, which is the process of recombination between genomic segments. Homologous recombination within segments is relatively (very) rare in the evolution of influenza A viruses (Nelson & Holmes 2007; Boni et al. 2008) and it is very unlikely that intra-segment recombination might take place during our one-year of study period (Aug 2009 – Aug 2010). Furthermore, recombinants in our sequence datasets would be identifiable as outliers in our root-to-tip analyses, as they would exist at the tips of long branches in the tree, and thus be more genetically distant from the root than expected given their sampling time. Therefore, we did not perform a recombination analysis for our alignments which is standard for the field (e.g., Nelson et al. 2008; Rambaut et al. 2008; Bedford et al. 2015), as there is no indication that one was necessary.

**Main comment 2**

**Comment:** I was wondering if the geographic origin of the virus expansion in China could be predicted, for example analyzing genetic diversity or ancestral sequence reconstruction, among other methods. And also, if possible, could be interesting to understand the spatiotemporal dynamics of the expansion from the genetic data, including geographic direction and speed of the expansion.

**Answer:** We agree with Dr. Arenas that identifying the geographic origins (as there are most certainly more than one entry point into such a large and diverse country) would be quite interesting. Indeed, previous studies have suggested that the origins of the A/H1N1pdm09 virus in China were the major cities of mainland China, such as Beijing and Shanghai (Bin et al., 2009; Liu et al., 2011), which is expected given typical pandemic virus epidemiology. Unfortunately, this type of combined phylogeographic analysis is beyond the scope of this study, as it would require many additional sequences from outside of China to contextualize the sequences from within China. This is because there are likely multiple introductions to China, and each of these introductions would require identification by including sequences from intervening geographic regions. Given this, a large phylogeographic study could be performed in a subsequent paper after we have resolved the movement of pandemic H1N1 *within* China during the early stages of the epidemic.

**Main comment 3**

**Comment:** The study should include additional previous studies about the spatial genetic structure of 2009 H1N1 influenza in some regions of China and compare with their results when possible.

**Answer:** While previous A/H1N1pdm09 studies in mainland China are often aspatial, analyzing the influenza trends within a single city or province, this study investigated the global patterns of the genetic structure across mainland China. Integrating findings from the local influenza trends and the genetic structure at a national scale would be challenging, as the spatial patterns of genetic characteristics of the virus may vary across the different spatial scales. Previous studies of 2009 H1N1 influenza were included in the first paragraphs of discussion.

**Main comment 4**

**Comment:** The study mentions the analysis of the population structure with the software STRUCTURE, however there is not any bar plot showing the estimated populations for every location.

**Answer:** The revised manuscript contains bar plots of HA and NA genes from the analysis of population structure in STRUCTURE, the estimated populations by province and by seven geographic regions (Supplementary figure 6).

**Main comment 5**

**Comment:** How the phylogenetic trees were rooted? This is fundamental to understand the evolutionary trajectories over time. It should be clarified in detail.

**Answer:** We used ‘A/California/04/2009’ as an outgroup sequence for rooting the ML phylogenetic trees, which is now clearly indicated in the methods. The Bayesian trees estimated in BEAST are automatically rooted using a molecular clock model informed by sampling times that imposes directionality on the tree.

**Main comment 6-1**

**Comment:** I missed a bootstrap analysis to evaluate the statistical support of the internal nodes of the reconstructed phylogenetic trees. If one aims to show a geographic structure, clades from different regions should be separated by nodes with high bootstrap values.

**Answer:** We included ML phylogenetic trees of HA and NA genes with the bootstrap supports of inner nodes greater than 50% as supplementary information. Importantly, we do not over-analyze the structure of the tree, but rather the pattern of pairwise distances between sequences along the tree, which is more robust to interpretation than the tree alone.

As we illustrated in maps, PCA plots, and Mantel’s correlograms, the geographic differentiation of genetic structure at regional or province scales is not apparent, and the findings from ML phylogenetic trees are consistent with these results. Only few internal nodes close to the tips have the bootstrap support greater than 50%, and the geographic differentiation of sequences by geographic region is not clear in phylogenetic trees as well (i.e., there are few monophyletic clades from specific regions of China). It is only in the analysis of the total matrix of pairwise distances that geographic patterns become apparent.

**Main comment 6-2**

**Comment:** It could be useful for readers to visualize the tree with clades for every geographic region (or for every cluster of Fig 5) presented with different colors.

**Answer:** The membership probabilities of HA obtained in STRUCTURE are not categorical but continuous, ranging from 0 to 1, thus it may not be appropriate to annotate these membership values in phylogenetic trees. Instead, we included ML trees with tips colored by geographic regions in supplementary information (Sup Fig 3 and 4) to see if the sequences from the same provinces tend to be in the same clades with the higher bootstrap supports.

**Main comment 7-1**

**Comment:** I missed a principal component analysis (PCA) for detecting clusters, note that PCA is frequently used for this purpose.

**Answer:** The PCA plots were included as supplementary information and the PCA analysis is now fully described in the methods section.

**Main comment 7-2**

**Comment:** Also, presenting FST estimates between populations from the same and from different clusters could be useful.

**Answer:** We calculated pairwise Fst estimates between provinces and reported as tables in supplementary information. In addition, maps of Fst estimates 1) between Hainan and top 5 closest provinces, 2) between Guangdong and top 5 closest provinces, and 3) between Shanghai and top 5 closest provinces were included as supplementary information.

**Main comment 8**

**Comment:** I found the discussion quite risky, with too much speculation trying to connect possible human migration events with the observed genetic structure. Many factors can affect the genetic structure, including local interactions, virus transmission and evolution, etc. I recommend being more cautious.

**Answer:** We revised the discussion sections to be more cautious about our interpretation of our findings. The Fst maps included as supplementary information may support our speculation about gene flows between populations.

**Minor comment 1**

**Comment:** I was wondering if the virus evolved following (or not following) a molecular clock. This can be explored with BEAST.

**Answer:** We used TempEst (v 1.5.3) to see if the evolutionary rates of HA and NA followed expected clock-like evolution, and it did.

**Minor comment 2**

**Comment:** I could not find the legends explaining what is shown in the figures of the manuscript.

**Answer:** The titles of legends were updated.

**References**

Bedford T, Riley S, Barr IG, Broor S, Chadha M, Cox NJ, Daniels RS, Gunasekaran CP, Hurt AC, Kelso A, Klimov A, Lewis NS, Li X, McCauley JW, Odagiri T, Potdar V, Rambaut A, Shu Y, Skepner E, Smith DJ, Suchard MA, Tashiro M, Wang D, Xu X, Lemey P, Russell CA. 2015. Global circulation patterns of seasonal influenza viruses vary with antigentic drift. Nature 523:217-220.

Bin C, Xingwang L, Yuelong S, Nan J, Shijun C, Xiayuan X, Chen W, National Influenza A Pandemic (H1N1) 2009 Clinical Investigation Group. Clinical and epidemiologic characteristics of 3 early cases of influenza A pandemic (H1N1) 2009 virus infection, People’s Republic of China, 2009. Emerging infectious diseases. 2009 Sep;15(9):1418.

Boni MF, Zhou Y, Taubenberger JK, Holmes EC. 2008. Homologous recombination is very rare or absent in human influenza A virus. Journal of Virology 82:4807-4811.

Liu Y, Wang W, Li X, Wang H, Luo Y, Wu L, Guo X. Geographic distribution and risk factors of the initial adult hospitalized cases of 2009 pandemic influenza A (H1N1) virus infection in mainland China. PloS one. 2011 Oct 12;6(10):e25934.

Nelson MI, Holmes EC. 2007. The evolution of epidemic influenza. Nature Reviews Genetics 8:196-205.

Nelson MI, Edelman L, Spiro DJ, Boyne AR, Bera J, Halpin R, Ghedin E, Miller MA, Simonsen L, Viboud C, Holmes EC. 2008. Molecular epidemiology of A/H3N2 and A/H1N1 influenza virus during a single epidemic season in the United States. PLoS Pathogens 4:e1000133.

Rambaut A, Pybus OG, Nelson MI, Viboud C, Taubenberger JK, Holmes EC. 2008. The genomic and epidemiological dynamics of human influenza A virus. Nature 453:615-619.
