# Supplementary material for: Spatial genetic structure of 2009 H1N1 pandemic influenza established as a result of interaction with human populations in mainland China
Source: PLoS One. 2023 May 17;18(5):e0284716. doi: 10.1371/journal.pone.0284716 (PMC10191359; doi:10.1371/journal.pone.0284716)
Supplement: Attachment — Submitted filename: Response to Reviewers.docx [file pone.0284716.s014.docx]

**Dear reviewers and Editors,**

We would like to express our gratitude for the comments and feedbacks on our manuscript. We appreciate the time and effort the editors and reviewers have taken to review our work, and we have carefully considered all of the suggestions and recommendations. Please find the revised manuscript and our answers based on the second reviewer’s comments below.

**Comment 1. Assuming the authors have some knowledge of the research context, but it may be helpful to provide more background information on the study design, methods, and objectives to help readers understand the research question and the significance of the results.**

**Answer:** We appreciate your suggestion. We revised the last paragraph of introduction to make clear about our study objectives, methods, and implication of the study results.

**Comment 2. Lines 425-457. The paragraph suggests that the movement of migrant workers is a potential driver of the observed genetic structure in influenza viruses. However, the evidence for this claim is somewhat circumstantial. The authors should clarify the strength of the evidence supporting this hypothesis and acknowledge other potential drivers of genetic structure.**

We should admit that this is one of the limitations of this study. As we were not able to obtain the population migration data during the first year of the 2009 pandemic in mainland China, the association between the periodic movements of migrant workers and the spatial patterns of genetic structure was not statistically tested. Instead, we included other previous studies investigating these relationships between migration and transmission in China, while clarifying the limitation of the study in the paragraph. As you suggested, we mentioned other potential factors driving viral spreading in the same paragraph.

**Comment 3. Is there any other similar research indicating that other viruses, especially RNA viruses, have transmission characteristics similar to the results of this study?**

Previous studies analyzed the geographic patterns of viral transmission and the association between human migrations and the spread of human infectious diseases in China, such as COVID-19 or STDs (e.g., Smith, 2005; Chen, et al., 2020; Wen et al., 2020; Zhan, et al., 2020). However, each virus has their own ecological and evolutionary characteristics to maintain circulation among human populations, thus spatial genetic structure of these virus may vary by viruses, environmental, or population characteristics (e.g., population immunity, population density, connectivity, and migration patterns). Moreover, these patterns may change over time as viruses evolve and adapt to enhanced human immunity from previous infections or vaccination, as we have seen in the COVID-19 cases. Therefore, we found that comparing the spatial patterns of the genetic structure of different viruses was not informative, instead, we focused on influenza A/H1N1pdm09 viruses in mainland China in this study.

**Reference**

Chen, Huijie, et al. "Correlation between the migration scale index and the number of new confirmed coronavirus disease 2019 cases in China." Epidemiology & Infection 148 (2020).

Charu, Vivek, et al. "Human mobility and the spatial transmission of influenza in the United States." PLoS computational biology 13.2 (2017): e1005382.

Smith, Christopher J. "Social geography of sexually transmitted diseases in China: Exploring the role of migration and urbanisation." Asia Pacific Viewpoint 46.1 (2005): 65-80.

Wen, Y., Wei, L., Li, Y., Tang, X., Feng, S., Leung, K., ... & Mei, S. (2020). Epidemiological and clinical characteristics of coronavirus disease 2019 in Shenzhen, the largest migrant city of China. MedRxiv, 2020-03.

Zhan, C., Tse, C. K., Fu, Y., Lai, Z., & Zhang, H. (2020). Modeling and prediction of the 2019 coronavirus disease spreading in China incorporating human migration data. Plos one, 15(10), e0241171.
